# Supplementary material for: An analysis of proteogenomics and how and when transcriptome-informed reduction of protein databases can enhance eukaryotic proteomics
Source: Genome Biol. 2022 Jun 20;23:132. doi: 10.1186/s13059-022-02701-2 (PMC9208142; doi:10.1186/s13059-022-02701-2)
Supplement: Supplementary file 4 — Additional file 4. Review history. [file 13059_2022_2701_MOESM4_ESM.docx]

**1^st^ round**

**Reviewer 1**

In their manuscript "Proteogenomics: an analysis of how and when it enhances proteomics" the authors provide an analysis of the effects of reducing the size of protein search databases based on gene expression evidence, with a focus on the effect on the false discorery rate (FDR) estimation, where smaller databases suffer from incorrect target-decoy modeling, and on protein inference.

Given the intense interest in proteogenomics, it is important to more precisely state what this paper provides and to differentiate it from proteogenomic approaches that aim to improve genome annotations of pro- and eukaryotes (i.e. identifying mainly small protein coding genes whose encoded products carry out an ever-increasing number of critical functions). For them, database size issues are also known to play a role, but for prokaryotes they can be overcome with efforts to limit the redundancy and including efforts like spectral re-scoring (PMID: 33109751). Combined with subsequent validation experiments they can provide independent evidence for the identification of a protein product encoded by a missed protein-coding gene (PMID: 29141959).

Here, the focus is different. The aim is to come up with an accurate list of protein identifications for eukaryotes that also capture variations compared to a reference database such as SNPs etc. observed for example in healthy or tumor samples, and which are based on available transcript data. For this, FDR cut-offs at the PSM level of 1% are ok (they would lead to too large number of false positives for the use case of finding novel protein-coding genes). Searching reference databases with post hoc filtering as preferable approach is an important message.

Comments

1. As is, the title is too broad, somewhat misleading and has to be changed. The manuscript (and the readers) will benefit from a title that more precisely captures its focus. The authors should reflect and limit it to the aspect of using proteogenomics for eukaryotes based on transcriptome-informed reduced databases. In prokaryotes, the genome of a strain under study can be readily de novo assembled; due to the reduced genome complexity and lack of splicing- even large proteogenomics search databases when searched with very stringent FDR control will lead to discovery of novelties that can be independently confirmed. Consequently, reduced transcriptome-based search databases in prokaryotes have only rarely been used following their initial description (PMID: 26072472), in stark contrast to the situation in eukaryotes.

2. In line with comment 1, more information should be provided in the introduction to differentiate this particular contribution from the original proteogenomics approach that aims to improve a genome annotation, i.e. that use e.g. proteomics or Ribo-seq data to provide evidence for as of yet unannotated gens that typically encode small proteins. This aspect of proteogenomics has also been receiving substantial and increasing interest over the last years. Notably, for this aspect, many of the implications discussed in the paper differ and a clarification will resolve this potential for mis-understandings.

3. A few more references to papers that describe several key concepts used should be added (mainly in Intro and Discussion), e.g. the major benefit of reduced protein databases is a benefit for protein inference as fewer shared peptides are present (line 42, Background). A few key papers/reviews should be cited for different protein inference approaches (probabilistic, deterministic, and bipartite graphs which are used, e.g. PMID: 20816881 in Intro; 20562346 and 29899466 in Discussion related to protein inference and quantification) that were developed to deal with shared peptides for protein inference and quantification. In L49; please add the reference for the "two peptide rule" (PMID: 15075378)

4. The abstract (L17) mentions "…when using excessively small databases". Can the authors further clarify and specify this statement, when they consider a database excessively small compared to the Ensembl reference protein DB? Furthermore, why is the "full DB size" in Figure 1 and FigS1 slightly different? I would expect the roughly 88000 entries to represent all 22400 gene models of EMBL GRHC38 and their possible splice forms (plus variants? -> unclear). Next, it is not clear how that number is then reduced to arrive at the roughly 23'000 entries; are these gene models and splice isoforms and additional variants (this should be specified)? This needs to be clearer for the readers given that the numbers e.g. from Ref 33 for the human lung dataset point at roughly 10-12000 expressed transcripts and proteins.

5. One limitation is that only two example datasets were analyzed in detail, which raises the question whether the conclusions can be broadly generalized as they are. Ideally, this should be addressed.

6. Another question concerns methodological aspects: the authors mention the search engine Mascot was used and that the data was post-processed with Proline (a relatively new search engine; see legend Figure 1). Were other search engines tested like MS-GF+ (PMID: 25358478) that have been shown to perform well with large DBs and in particular for proteogenomics and that identify many more PSMs than Mascot at a chosen FDR cut-off?

7. The information which splice isoforms can be identified by a peptide is indeed very relevant. It could be used to exclude shared peptides from the protein inference step or to distinguish between shared peptides that imply proteins encoded by different gene models from shared peptides that imply several isoforms encoded by one gene model (which is more informative). This concept was introduced a while back (PMID: 20622826) and, as the percentage of shared peptides is rather high for eukaryotes, could be useful.

8. While not very common, it has been described in the literature that for genes not expressed above a given cut-off, the encoded protein still could be identified (can e.g. arise for genes located in duplicated regions, depending on how multiply mapping reads are handled). This could be mentioned in the Discussion as another limitation of a reduced search DB. In this case, and in contrast to proteins not being identified from highly expressed genes (which would make it into a limited database), the protein sequences would be missing from the database resulting in false negatives. Also, the consideration of spectral re-scoring and concepts to include predicted ion intensities (PMID: 31133760), which is gaining momentum, would be good to add again in the discussion.

**Reviewer 2**

The authors present an interesting study focused on assessing the value of integrating transcriptome sequencing information with mass spectrometry‐based shotgun proteomics data (an approach generally called proteogenomics). This integrative analysis has been utilized for a number of years to increase the depth of information that can be gleaned from MS‐based proteomics. However, as the authors correctly point out, there are some potential pitfalls in such an approach, necessitating caution in how it is applied. The authors focus on two main applications of integrative proteogenomics: 1) Using transcriptome sequencing data to create a reduced protein sequence database based on mRNA expression and assumed translation of proteins; and 2) The use of transcriptome sequencing data to assist with protein inference. Both of these applications utilize tandem mass spectrometry (MS/MS) data generated from complex protein mixtures, which is then matched against sequences within the protein database to identify peptides and infer proteins present within the sample. The main findings of the work are that compared to using traditional methods, the reduced transcript‐guided protein database generates inflated false‐positives peptide spectrum matches (PSMs); and secondly, that incorporating transcript sequences can improve the accuracy of inferring proteins from PSMs derived from MS/MS data. The authors do provide some interesting insights into the use of alternative statistical analysis methods (e.g. Benjamin‐Hochberg) for controlling false positive matches in reduced sequence databases, and they provide an R‐script that may be useful for improving accuracy of protein inference when integrating transcriptomic and proteomic information. That being said, as currently presented the work only has limited novelty, as the conclusions they reach have been described previously. This dampens enthusiasm for the work overall.

Main comments

1. As the authors correctly point out, the use of sequence databases that are reduced in size increases false positive PSM potential. Although for a slightly different application (metaproteomics), the authors point out that it has been shown that reduced databases inflate false positives (e.g. see Proteomics 2015 Oct;15(20):3439‐53; and J Proteome Res. 2020 Jul 2;19(7):2772‐2785). Given these past descriptions, what is novel about their findings? Perhaps the most interesting part of these findings is that BH statistical analysis might be a remedy for this inflation of false positives, but this is somewhat of a secondary focus on the paper. The use of transcriptomic sequencing to decrease ambiguity of protein inference from MS/MS data has also been described (e.g. see J Proteome Res. 2012 Feb 3;11(2):1009‐17). It is unclear how their work is providing new insights here, beyond the R script that has been developed?

2. The authors ignore one of the main applications of proteogenomics – which seeks to provide evidence of expression of proteins with novel amino acid sequences that are indicated by the transcriptomic sequencing data. Here, the standard full sequence database is used and possible novel sequences are appended (or possibly searched sequentially using separate database for references and variant sequences). There are many unanswered questions in this approach related to the reliability of PSMs identifying variant sequences – an evaluation of this area of work would be very valuable. Could they apply new statistical filters in order to better ensure matches to variant sequences are reliable? This seems like a missed opportunity in the work.

3. No mention is made of the role of post‐translational modifications (PTMs) in the PSMs that come from transcript‐guided databases and PSM generation. How would inclusion of PTMs to peptides potentially alter results?

Minor points

1. A smaller point related to the use of transcript abundances to infer proteins. What are some limitations of the assumption that all proteins identified should have detectable transcripts? Given differences between protein and mRNA half‐lives, one could imagine some long‐lived proteins being present that do not have detectable transcripts. Does this represent a downside? Additionally, integrating transcript sequencing data could be valuable in discerning between protein isoform expression – does their script enable this analysis?

2. Overall the data is nicely presented. However, Figure 3A does not have axes labelled

**Reviewer 3**

This manuscript addresses the persistent misunderstanding of the field of the proper uses of FDR control in proteogenomics database searches. The authors demonstrate clearly that it is a mistake to associate an increase in proteomics search sensitivity with a decrease in database size. Rather, this is a statistical error due to a flaw in target decoy comparisons (TDC). They demonstrate that reducing the database size can result in insufficient number of decoys to simulate incorrect target matches accurately, causing lower scoring cutoffs for the same false discovery rate (FDR). They also show that the Benjamini-Hochberg (BH) procedure produces more conservative and stable FDR control than does TDC. They then demonstrate that reduced database searches with BH-based FDR control yield smaller protein groups and protein identifications with less ambiguity. Finally, the authors use an alternative strategy that consists of a full database tandem MS search with post-hoc removal of proteins for which no transcript was expressed, and no peptide identified. This reduces the number of ambiguous protein identifications that result from shared peptides. In summary, if this work is read and understood widely, there will be an improvement in the appropriate use of proteogenomics data in the field.

**Authors’ response**

We would like to first thank the Editor and the three Reviewers for their involvement as well as their advices to improve our manuscript. We found all their remarks insightful, and we were able to positively account for them in this revised version of our manuscript. **Concretely, this has led us to:**

- **Better underline the novel aspects of this study** and its impact, even beyond transcriptome-informed reduced database searches. In particular:
  - The impact of TDC instability with respect to database size for **novel peptides identification** (Reviewer 2 Main Comment 2) or **PTMs identification** (Reviewer 2 Main Comment 3)**, where BH application can be of help** (Reviewer 2 Main Comment 1.A);
  - The advantage of a transcriptome-informed post-hoc filtering approach over a reduced database search (a novelty apparently unnoticed by Reviewer 2, see our comment below).
- **Perform extensive complementary evaluations, with additional datasets** (Reviewer 1 Comment 5) **and search engines** (Reviewer 1 Comment 6).
- **Complete the Discussion section with a paragraph about protein expressed without transcriptomic evidence** (Reviewer 1 Comment 8; Reviewer 2 Minor Point 1.A). **Likewise, it has led us to provide additional elements concerning the capability to discriminate between isoforms based on shared peptide sequences** (Reviewer 1 Comment 7; Reviewer 2 Minor Point 1.B).
- **Better account for the state-of-the-art and cite the references suggested by all Reviewers** (Reviewer 1 General Comment, Comments 1, 3, 7 and 8; Reviewer 2 Main Comments 1.A and 1.B).
- **Improve the focus of the paper with a sharper introduction and a new title** (Reviewer 1 General Comment, Comments 1 and 2).
- **Check and correct the figures pointed by the reviewers** (Reviewer 1 Comment 4.2, Reviewer 1 Comment 4.3; Reviewer 2 Minor Point 2).

In addition, we have discussed in the rebuttal below the more open question raised by Reviewer 1 (Comment 4.1) about the adequate size of the decoy database.

**Concerning the novelty of our work:** Like the Editor, we have noticed Reviewer 2’s concerns. We agree with the Editor that based on how these novelties have been summarized, our manuscript does not appear as novel. However, we believe that Reviewer 2 was misled by our presentation and phrasing, and we apologize for this. We did our best to clarify this, both in the revised version and in our response to Reviewer 2’s comments (see below). Hereafter, we briefly explain why our work is more novel than originally perceived by Reviewer 2:

- Reviewer 2 pinpointed that previous works in metaproteomics [PMID:27671352; PMID:32396365] have already described that “*reduced transcript‐guided protein database generates inflated false‐positives PSMs*”. Although this description is not universally shared (as noted by both Reviewers 1 and 3), we acknowledge its existence in previous literature. However, we have added the following elements of novelty: (i) **Our work provides a mechanistic explanation for this** (which does not appear in [PMID: 27671352] or in [PMID: 32396365]); (ii) Our work demonstrates that false positive inflation is **not inherent to the database size (as oversimplified in literature or by Reviewer 2), but to the statistical estimate used to control the FDR, which opens room for statistically correct use of reduced database**. Therefore, we believe our work to shed two interesting lights on this question.
- Reviewer 2 pointed [PMID:22103967], which mentions the interest of reduced database to limit ambiguities in protein identification. However, **its authors incorrectly explained this by a supposed increment of true positive PSMs** (which ironically enough, violates the point discussed above), as this would provide more information to discriminate ambiguous protein identifications. In this work, we were able to improve protein identification likewise, while correctly controlling the FDR (i.e., without assuming that additional false-positive PSMs were correct). Moreover, we were able to **precisely quantify and describe the extent to which protein identification is improved**. Thus, our work goes beyond this reference in many aspects (this is also why [PMID:22103967] was already cited in the first version of our manuscript).
- Finally, Reviewer 2 obliterated our proposal to replace a priori database reduction by **post-hoc filtering**. Thanks to our quantification of “pure reallocations” (traditionally assumed majority, while they are in fact minority), we could demonstrate that post-hoc filtering indeed yielded similar performances. However and in contradiction with database reduction, it provides a transparent methodological workflow, which, as acknowledged by Reviewers 1 and 3, is of prime interest to limit the statistical flaws that classically hamper the quality of proteogenomics experiments. To the best of our knowledge, this has never been proposed so far.

**Therefore, we believe our work is more novel than originally perceived by Reviewer 2**. In this revised version, we better emphasized these findings. Nonetheless, we found Reviewer 2 suggestion over the potential impact of our findings on the identification of novel/variant peptides or of PTMs very insightful. Although an in-depth exploration of the issues related to novel peptide and/or PTM identifications are beyond the scope of this article, we found it important to underline how TDC instability with respect to database size also affects them; as well as how the application of an alternative method of FDR control than TDC, such as Benjamini-Hochberg adjustment, can be of help in that context. Therefore, in the revised version of the manuscript we added a paragraph discussing these aspects.

Together with the various other improvements suggested by the three reviewers and summarized above, this has led us to a deeply revised version, which we hope to fulfill the Reviewers’ and the Editor’s expectations.

Reviewer 1

In their manuscript "Proteogenomics: an analysis of how and when it enhances proteomics" the authors provide an analysis of the effects of reducing the size of protein search databases based on gene expression evidence, with a focus on the effect on the false discorery rate (FDR) estimation, where smaller databases suffer from incorrect target-decoy modeling, and on protein inference. Given the intense interest in proteogenomics, it is important to more precisely state what this paper provides and to differentiate it from proteogenomic approaches that aim to improve genome annotations of pro- and eukaryotes (i.e. identifying mainly small protein coding genes whose encoded products carry out an ever-increasing number of critical functions). For them, database size issues are also known to play a role, but for prokaryotes they can be overcome with efforts to limit the redundancy and including efforts like spectral re-scoring (PMID: 33109751). Combined with subsequent validation experiments they can provide independent evidence for the identification of a protein product encoded by a missed protein-coding gene (PMID: 29141959). Here, the focus is different. The aim is to come up with an accurate list of protein identifications for eukaryotes that also capture variations compared to a reference database such as SNPs etc. observed for example in healthy or tumor samples, and which are based on available transcript data. For this, FDR cut-offs at the PSM level of 1% are ok (they would lead to too large number of false positives for the use case of finding novel protein-coding genes). Searching reference databases with post hoc filtering as preferable approach is an important message.

>> We thank Reviewer 1 for this detailed summary of our approach as well as of our results. We also thank Reviewer 1 for their suggestion to clarify the focus of our investigations. In the introduction of the revised version of the manuscript, we have added the suggested references, which helped us stress the distinction between the various types of proteogenomics investigations, both about the objective (genome annotation vs. proteomics improvement) and about the genome complexity (prokaryotes vs. eukaryotes).

Comments

1.	As is, the title is too broad, somewhat misleading and has to be changed. The manuscript (and the readers) will benefit from a title that more precisely captures its focus. The authors should reflect and limit it to the aspect of using proteogenomics for eukaryotes based on transcriptome-informed reduced databases. In prokaryotes, the genome of a strain under study can be readily de novo assembled; due to the reduced genome complexity and lack of splicing- even large proteogenomics search databases when searched with very stringent FDR control will lead to discovery of novelties that can be independently confirmed. Consequently, reduced transcriptome-based search databases in prokaryotes have only rarely been used following their initial description (PMID: 26072472), in stark contrast to the situation in eukaryotes.

>> This suggestion is in line with the previous comment, and we followed it likewise. The suggested reference was also added in the introduction. Finally, the title now reads: **Transcriptome-informed reduction of protein databases: an analysis of how and when proteogenomics enhances eukaryotic proteomics**. We thank Reviewer 1 for these suggestions.

2.	In line with comment 1, more information should be provided in the introduction to differentiate this particular contribution from the original proteogenomics approach that aims to improve a genome annotation, i.e. that use e.g. proteomics or Ribo-seq data to provide evidence for as of yet unannotated gens that typically encode small proteins. This aspect of proteogenomics has also been receiving substantial and increasing interest over the last years. Notably, for this aspect, many of the implications discussed in the paper differ and a clarification will resolve this potential for mis-understandings.

>> We agree with Reviewer 1, and as previously responded, we have modified the manuscript accordingly, adding a paragraph and references in the introduction.

3.	A few more references to papers that describe several key concepts used should be added (mainly in Intro and Discussion), e.g. the major benefit of reduced protein databases is a benefit for protein inference as fewer shared peptides are present (line 42, Background). A few key papers/reviews should be cited for different protein inference approaches (probabilistic, deterministic, and bipartite graphs which are used, e.g. PMID: 20816881 in Intro; 20562346 and 29899466 in Discussion related to protein inference and quantification) that were developed to deal with shared peptides for protein inference and quantification. In L49; please add the reference for the "two peptide rule" (PMID: 15075378)

>> We thank Reviewer 1 for this suggestion about literature exhaustiveness. We have added all the pointed references in the various sections suggested by Reviewer 1, as well as [PMID: 22103967] (in the Background section, nearby line 42 in the original version, and at line 58 in the revised version).

4.1.	The abstract (L17) mentions "…when using excessively small databases". Can the authors further clarify and specify this statement, when they consider a database excessively small compared to the Ensembl reference protein DB?

>> This is a question we recurrently faced when presenting our work in conferences. We agree with Reviewer 1, as it would actually be of real interest to be able to quantify when the database is large enough for TDC to yield accurate FDR, and when it is not. Unfortunately, our past and current investigations suggest that it is not possible to give numbers, mainly because TDC is not only sensitive to the database size, but also to various other parameters, including the precursor and fragment mass tolerance sets in the MS, as well as the shuffling process generating decoy sequences. For this latter aspect, theoretical works in high-dimensional statistics are insightful, but still largely non-conclusive. Notably, thanks to [https://doi.org/10.1111/rssb.12265], it is now possible to build a quality control criterion on the decoy generation (termed “knock-offs” or “mirror variables” in statistics). However, this criterion is only expressed in algebraic terms (the shrinked correlation structure of a pair of ill-conditioned matrices) and attempts to connect it to an algorithmic procedure that shuffles amino acid sequences is only an emerging subject [https://arxiv.org/abs/1907.01458].

4.2.	(Furthermore, why is the "full DB size" in Figure 1 and FigS1 slightly different? I would expect the roughly 88000 entries to represent all 22400 gene models of EMBL GRHC38 and their possible splice forms (plus variants? -> unclear).

>> We thanks Reviewer 1 for noticing this. After double-check, it appears the numbers are correct, but the way we presented them misled Reviewer 1. In the revised version, we modified Figure 1 and Figure S1 to make it clear that the total number of proteins in full DB is the sum of those also included in the reduced DB (e.g., 22,786 for Jurkat in Figure 1) and those not included in the reduced DB but only contained in the full DB (e.g., 88,002 for Jurkat in Figure 1). These two numbers in the Venn diagrams sum up to 110,788 for human samples and up to 67,158 for the mouse sample, in both Figure 1 and Figure S1. To make it clearer we added to the figures the total number proteins next to “full DB”.

Here, the total number of proteins represents all splice forms but not variants, and this has also been clarified in the in the “Construction of reduced transcriptome-informed protein databases for MS/MS searches” section of Methods, in the revised text.

4.3.	Next, it is not clear how that number is then reduced to arrive at the roughly 23'000 entries; are these gene models and splice isoforms and additional variants (this should be specified)? This needs to be clearer for the readers given that the numbers e.g. from Ref 33 for the human lung dataset point at roughly 10-12000 expressed transcripts and proteins.

>>This number represents all splice forms but not variants. This has been clarified in the Results section and in the Methods section of the revised text. Numbers reported from former Reference 33 (now reference 43) for the human lung dataset represent instead the number of genes detected on protein and transcript level, which explains the difference.

5.	One limitation is that only two example datasets were analyzed in detail, which raises the question whether the conclusions can be broadly generalized as they are. Ideally, this should be addressed.

>> We added two additional datasets in order to verify whether our conclusions could be easily generalized. One dataset was generated from a human healthy spleen tissue, the other from mouse epithelial colon cells sorted by flow cytometry. The four samples significantly vary in number of spectra. Many more proteomic spectra are available for Spleen and Lung, as 36 fractions were obtained by hSAX off-line chromatography and used for bottom-up mass-spectrometry based proteomics. The mouse dataset, instead, represents a case with little availability of starting material, as this was a particular type of mouse epithelial cells, isolated by flow cytometry. Moreover, the datasets were generated with different protocols and in different laboratories. Overall, these four datasets are quite diverse. As it appears in the manuscript, the results on the two newly added datasets concur with those obtain on the two original datasets, which makes us confident on the generalization capabilities of the reported conclusions.

6.	Another question concerns methodological aspects: the authors mention the search engine Mascot was used and that the data was post-processed with Proline (a relatively new search engine; see legend Figure 1). Were other search engines tested like MS-GF+ (PMID: 25358478) that have been shown to perform well with large DBs and in particular for proteogenomics and that identify many more PSMs than Mascot at a chosen FDR cut-off?

>> We thank Reviewer 1 for spotting the lack of variety in the search engine. To cope for this, we have duplicated our experiments with the pipeline suggested by Reviewer 1, namely, MS-GF+. In a nutshell, we observed results that concurred with our previous analyses:

- First, on the reduced database, all the spectra that matched on a target sequence in the reduced database did so with a score equal to or lower than the score obtained in the full database (either target or decoy, see supplemental data Additional File 1: Table S3). In other words, additional identifications in the reduced database do not come from an improved search score of target matches in the reduced database.: the matching process in the reduced database cannot be assumed more sensitive.
- Second, by comparing the TDC cutoffs at various FDR thresholds (0.5%, 1% and 5%; see supplemental data Additional File 1: Table S5) in both the full and reduced databases, we observed the same decrease of the cutoffs as with Mascot.

These two pieces of evidence support our finding about the instability of TDC with reduced database. However, it was not possible to extend the other results of the paper to the MS-GF+ pipeline, namely the comparison with BH-based FDR control, and the subsequent protein inference. Indeed, BH correction cannot be directly applied to search engine results which score’s formula is not sufficiently detailed (in mathematical terms), including MS-GF+ (but also, e.g., Andromeda/MaxQuant). The reason is that p-values are required as input to BH and they cannot be reverse-engineered in absence of sufficient information.

Fortunately, our findings (namely, (i) the absence of reallocation on a target with a higher score in the reduced database than the original match in the full database search, either target or decoy; (ii) the reduction of the cutoffs when shifting from a full database to a reduced one) are sufficient to asses our conclusions about TDC sensitivity generalize well.

7.	The information which splice isoforms can be identified by a peptide is indeed very relevant. It could be used to exclude shared peptides from the protein inference step or to distinguish between shared peptides that imply proteins encoded by different gene models from shared peptides that imply several isoforms encoded by one gene model (which is more informative). This concept was introduced a while back (PMID: 20622826) and, as the percentage of shared peptides is rather high for eukaryotes, could be useful.

>> We thank Reviewer 1 for this comment, which we agree with. As illustrated on Figure 4E, reduced database searches do not only lead to a smaller number of shared peptides between splice isoforms of the same genes, but also between homologous proteins from different genes. Our code (now an R package available on CRAN) does not automatically classify shared peptides between different isoforms vs. different genes, which is already implemented in the PeptideClassifier software (from the suggested reference [PMID:20622826], now included in the Introduction). Rather, it allows to visually inspect each group of proteins connected by shared peptides: for any particular protein of interest, a bipartite (peptide-protein) graph can be generated showing which and how many proteins it shares the peptide(s) with. Given the protein identifiers, the user can find out if it shares peptide(s) with isoforms of same gene, with proteins from different genes or with both.

For the same protein, these graphs can be generated before and after performing transcriptome-informed filtering of proteomic identifications. In this way, the effect of the filtering performed to aid protein inference is completely transparent and easy to interpret by the user.

Furthermore, different options are available for the transcriptome-informed filtering strategy, which affect how shared peptides are treated. The first option (not recommended) is to remove proteins with no transcript expression and the peptides only mapping on these proteins (either specific or shared by these proteins only). The second option is to remove proteins satisfying all the following criteria: no transcript expression and no specific peptides; then remove all peptides only shared between the removed proteins. The third option is to remove proteins satisfying all the following criteria: no transcript expression, no specific peptides and all their peptides being shared with at least one of the retained proteins, so that all peptide identifications are kept and only proteins are filtered out.

8.	While not very common, it has been described in the literature that for genes not expressed above a given cut-off, the encoded protein still could be identified (can e.g. arise for genes located in duplicated regions, depending on how multiply mapping reads are handled). This could be mentioned in the Discussion as another limitation of a reduced search DB. In this case, and in contrast to proteins not being identified from highly expressed genes (which would make it into a limited database), the protein sequences would be missing from the database resulting in false negatives. Also, the consideration of spectral re-scoring and concepts to include predicted ion intensities (PMID: 31133760), which is gaining momentum, would be good to add again in the discussion.

>> We agree with Reviewer 1 on this point. We commented on this limitation of reduced database searches in the Discussion and highlighted that this limitation is partially overcome by the transcriptome-informed filtering strategy we propose. More in general, an intrinsic limitation of database searches in proteo(geno)mics is that one cannot work under the open world assumption: only protein sequences which are in the database can be identified. To outstep this limitation, De Novo scoring has long been identified, but after 10 to 15 years, the performances are still below the expectations. In this context, spectral rescoring can indeed provide an in-between solution (in the sense that it does not entirely fulfill the De Novo objectives while still extending classical database search capabilities). Our personal feeling is that in the future, the techniques underlying these approaches (deep architectures and transformer models) will improve to the point they will be mature enough to directly tackle the challenge of De Novo sequencing. Following the advice of Reviewer 1 (for which we are grateful of), we have added a paragraph about this in the Discussion section.

Reviewer 2

The authors present an interesting study focused on assessing the value of integrating transcriptome sequencing information with mass spectrometry‐based shotgun proteomics data (an approach generally called proteogenomics). This integrative analysis has been utilized for a number of years to increase the depth of information that can be gleaned from MS‐based proteomics. However, as the authors correctly point out, there are some potential pitfalls in such an approach, necessitating caution in how it is applied. The authors focus on two main applications of integrative proteogenomics: 1) Using transcriptome sequencing data to create a reduced protein sequence database based on mRNA expression and assumed translation of proteins; and 2) The use of transcriptome sequencing data to assist with protein inference. Both of these applications utilize tandem mass spectrometry (MS/MS) data generated from complex protein mixtures, which is then matched against sequences within the protein database to identify peptides and infer proteins present within the sample.

>> We thank Reviewer 2 for this accurate summary of the methodological and applicative focuses of our work.

The main findings of the work are that compared to using traditional methods, the reduced transcript‐guided protein database generates inflated false‐positives peptide spectrum matches (PSMs); and secondly, that incorporating transcript sequences can improve the accuracy of inferring proteins from PSMs derived from MS/MS data. The authors do provide some interesting insights into the use of alternative statistical analysis methods (e.g. Benjamin‐Hochberg) for controlling false positive matches in reduced sequence databases, and they provide an R‐script that may be useful for improving accuracy of protein inference when integrating transcriptomic and proteomic information. That being said, as currently presented the work only has limited novelty, as the conclusions they reach have been described previously. This dampens enthusiasm for the work overall.

>> We hereby understand that the original version of our article did not sufficiently highlight the novelties of our work, and we apologize for this. This has been largely accounted for in the revised version of the manuscript, but to facilitate the scientific discussion, we hereafter explain why our work is indeed very novel in many aspects:

1. According to Reviewer 2, the first main finding of our work is that “*the reduced transcript‐guided protein database generates inflated false‐positives PSMs*”. **However, our work demonstrates this very formulation does not hold**: although the false positive inflation indeed occurs, it is not generated by the database reduction, but by the use of an FDR control method (namely TDC) that is shown to be unstable with respect to the database size. **To the best of our knowledge, clearly showing that the bottleneck lies in the FDR estimate rather than in the database size and exhibiting in a very explicit way the mechanism by which it occurs is a real novelty**. We believe that this novelty is relevant for the proteogenomics community for two reasons. First, it encourages discussion on the usage of methods of FDR control alternative to TDC (e.g., BH), which can avoid TDC flaws with reduced databases. Second, our clear and detailed explanation of the issue helps to increase well-informed recognition of this issue. Indeed, there still is limited community awareness about the risks of a combining TDC and reduced databases. This limited awareness is confirmed by Reviewer’1 and Reviewer’s 3; but also by the authors of [PMID: 22103967] (a reference suggested by Reviewer 2 in Main Comment 1). Notably, these authors claimed the use of a reduced database yielded improved sensitivity and also seemed to ignore their reported protein inference improvement was not due to such improved sensitivity at peptide level (we showed in our work that we can reduce ambiguity of protein identifications without this artifactual increase of identifications). Increased sensitivity from searching transcriptome-informed protein databases, followed by TDC FDR control was also claimed in [PMID25149441: Sheynkman, Gloria M., et al. "Using Galaxy-P to leverage RNA-Seq for the discovery of novel protein variations." BMC genomics 15.1 (2014): 1-9.], which proposes a Galaxy workflow to exploit this. Other publications also propose to reduce database size based on transcriptome information and do not comment on TDC sensitivity to database size (e.g*.*, [PMID25263569: Woo et al. Proteogenomic strategies for identification of aberrant cancer peptides using large-scale Next Generation Sequencing data]; [PMID26569054: Shanmugam and Nesvizhski Effective leveraging of targeted search spaces for improving peptide identification in MS/MS based proteomics. J Proteome Res 2015]), so that globally, their main message seems to be that the smaller the database, the higher the number of identifications.
    In this context, we did not want to formulate our results so that it could be interpreted as a “reduced-database blank check”. We believe proteogenomics practitioners to be less responsive to a message like “it is possible to compensate for a false positive inflation you may not be aware of, by using alternative statistical methods that you are not a regular user of” than to a message like “your classical pipeline made of reduced database and TDC based FDR tends to produce false positives, so if you really need reduced database, it is important to change your statistical tools”. We assume this in-between message was more blurring than anything else to Reviewer 2 (who is obviously more aware of these pitfalls than the average proteogenomics researcher) and we apologize for this. We acknowledge that our original formulation was not sufficiently clear, and we did our best to improve it in the revised version.
2. According to Reviewer 2, our second main finding only amounts to an R script (see also Reviewer 2’s Main Comment 1 below). We disagree with this view. Although we hope this script (at the time of submission only available on GitHub but now also available as an R package on CRAN) to be useful to the proteogenomics community, our manuscript is more insightful. First, and contrarily to previous literature (see our response to Reviewer 2’s Main Comment 1 below), we were able to exhibit an improvement in protein identification without relying on erroneous statistical filters (as well as erroneous mechanistic explanations). Second, we provided a detailed and quantitative description of how protein ambiguity is reduced. Moreover, to do so, we had to rely on a peptide-centric view of peptide grouping (formalized through the well-known notion of connected components in the peptide-protein graph) that proved to be an interesting alternative to the traditional protein-centric view.
3. Finally, Reviewer 2 did not mention a third novelty: we proposed to replace the preliminary database reduction by post-hoc transcriptome-informed filtering. Thanks to our quantification of “pure reallocations” (traditionally assumed majority, while they are in fact minority), we could demonstrate that post-hoc filtering indeed yielded similar performances. However and in contradiction with database reduction, it provides a transparent methodological workflow, which as acknowledged by Reviewers 1 and 3, is of prime interest to limit the statistical flaws that classically hamper the quality of proteogenomics experiments. This post-hoc filtering strategy is also implemented in the provided R package: more details and critical comments on its usage have been added in Methods and in the Discussion of the revised version.

Our revised version of the article makes all these novelties more explicit. We hope Reviewer 2 will find these improvements suited.

Main comments

1.A	As the authors correctly point out, the use of sequence databases that are reduced in size increases false positive PSM potential. Although for a slightly different application (metaproteomics), the authors point out that it has been shown that reduced databases inflate false positives (e.g. see Proteomics 2015 Oct;15(20):3439‐53; and J Proteome Res. 2020 Jul 2;19(7):2772‐2785). Given these past descriptions, what is novel about their findings? Perhaps the most interesting part of these findings is that BH statistical analysis might be a remedy for this inflation of false positives, but this is somewhat of a secondary focus on the paper.

>> We thank Reviewer 2 for these references we were not aware of. Despite a different application, we believe them to be related to our subject of investigation, and we have added them to the revised version of the manuscript (more precisely in the Introduction). However, after scrutinizing them, we do believe our work goes beyond them and proposes new insights. Both papers indeed shared the same concern (namely, changing the database size with the current statistical workflow yields questionable FDR control), however, none of them proposed a convincing explanation of why such a pitfall occurs, clearly showing, as we instead did, that the problem does not lies in the database reduction, but in the TDC procedure. We believe that there is a huge difference between describing a problem in qualitative terms (as done in elder literature) and providing a quantitative description together with workaround solutions (as we did). We agree with Reviewer 2 about the application of BH procedure. Because it is conceptually simple and easy to apply, we anticipate it to be of real practical interest for the community. Yet, as noted by Reviewer 2, we did not put the main focus of the paper on it for two reasons: First, we believe that in scientific literature, the mere transposition of a now standard statistical tool to a new problem should not be described with too much superlatives. Second, in omics sciences, the need for well-defined statistical guidelines tends to make things a bit too binary (either “mandatory” or “incorrect/forbidden”), while statistical reality is a bit more nuanced: We do not want our readership to over-interpret our results. Although TDC is unstable, it can be interesting in some cases. BH is a good alternative (which we hope will spread in the community) but it is not the only one (e.g., empirical null estimation [doi.org/10.1198/016214501753382129], Target-Decoy without competition [PMID: 18067246], Bayesian FDRs [PMID: 27756721], etc.). For all these reasons, we believe it is wiser to keep the focus of the paper on the concrete novelties we have summarized above.

1.B	The use of transcriptomic sequencing to decrease ambiguity of protein inference from MS/MS data has also been described (e.g. see J Proteome Res. 2012 Feb 3;11(2):1009‐17). It is unclear how their work is providing new insights here, beyond the R script that has been developed?

>> This article [PMID: 22103967] was already referenced and cited in the original version of the article, and we believe our work significantly improves upon it. We acknowledge it contains a description of the ambiguity decrement of protein inference when using reduced databases. However, it reaches this conclusion by relying on two erroneous arguments. First, the authors seem to ignore that using TDC with reduced database makes the FDR unreliable, so that they do not consider the inflation of false-positive PSM at a given FDR cutoff and instead, erroneously conclude that reduced database increases sensitivity. Second, they perform protein inference using a parsimony-based algorithm and assume that the reduced ambiguity of protein identifications is a direct consequence of the increased sensitivity at peptide level (in a nutshell, with more validated PSMs, one increases the likelihood to identify specific peptides, from which a more parsimonious protein group can be reported).

On the contrary, we have demonstrated that a similar reduction in ambiguity of protein identifications could be obtained even with a strict FDR control (i.e., without artificially increasing the number of validated PSMs with false positives, i.e., at broadly constant number of PSMs). Moreover, we were able to precisely describe and quantify this ambiguity reduction and we measured it prior to any protein inference step (such as parsimony-based algorithm) to clearly show that it was a direct consequence of the reduced database search, independent of any downstream approach of protein inference.

Together with the connected component analysis, the R package (acknowledged by Reviewer 2) and the post-hoc filtering methodology (see our answer above, as well as comments from Reviewers 1 and 3) we believe that our manuscript presents a significant state-of-the-art improvement with respect to [PMID: 22103967].

2. The authors ignore one of the main applications of proteogenomics – which seeks to provide evidence of expression of proteins with novel amino acid sequences that are indicated by the transcriptomic sequencing data. Here, the standard full sequence database is used and possible novel sequences are appended (or possibly searched sequentially using separate database for references and variant sequences). There are many unanswered questions in this approach related to the reliability of PSMs identifying variant sequences – an evaluation of this area of work would be very valuable. Could they apply new statistical filters in order to better ensure matches to variant sequences are reliable? This seems like a missed opportunity in the work.

>> We thank Reviewer 2 for this comment. Indeed, when searching peptide variants, similar issues (target-decoy sensitivity to database size) can be encountered. Peptide variants have higher false positive identification rates than novel peptides and for this reason, it was proposed to identify them using separate searches for variant and canonical peptides [PMID31031003: Vasaikar et al. Proteogenomic analysis of human colon cancer reveals new therapeutic opportunities. Cell 2019]; [PMID17488105: Bunger et al. Detection and validation of non-synonymous SNPs from orthogonal analysis of shotgun proteomics data. J Proteome Res. 2007] or using a common search followed by separate FDR validation [PMID21389108: Li et al. A bioinformatic workflow for variant peptide detection in shotgun proteomics. MCP. 2011]; [PMID25357241: Nesvizhskii. Proteogenomics: concepts, applications and computational challenges. Nat methods 2014]; [PMID24240322: Branca et al. HiRIEF LC-MS enables deep proteome coverage and unbiased proteogenomics. Nat Methods. 2014]. However, these strategies may cause the number of decoys for the validation of variant peptides to be too small for accurate FDR control, when using the conventional target-decoy approach. This is why additional filters or modifications of the target-decoy protocol for FDR control were proposed to avoid this [PMID: 17488105]; [PMID: 21389108]. However, there is still no consensus on whether we should append the canonical and variant protein databases and perform a single validation or rather search and validate them separately, which impairs fair comparison between studies. Worse, not all these studies verify the assumptions for correct application of target-decoy FDR control and try to circumvent these issues. This is why we think that our work can raise awareness about it. We thank Reviewer 2 for the comment, which can be indeed very useful for the community. We added a paragraph in the Discussion to explain how the issue of TDC sensitivity to database size also applies to variant peptide identification. However, we did not perform any further experiment because: (*i*.) Applying the proposed BH alternative for FDR control to variant peptide identification from separate searches would not add new information but would only be an example of application. (*ii*.) Investigating more deeply this subject (for example the impact of relative frequency of variants compared to canonical peptides, or the issue of amino acid substitutions with same mass shift as PTMs, etc.) is beyond the scope of this manuscript and corresponds to another field of research *per se*.

3. No mention is made of the role of post‐translational modifications (PTMs) in the PSMs that come from transcript‐guided databases and PSM generation. How would inclusion of PTMs to peptides potentially alter results?

>> With this comment, Reviewer 2 points a very interesting yet complex question. As it occurs in proteogenomics, one of the main challenges of PTM identification is the size of the search space, which increases even faster than in proteogenomics. Indeed, while in proteogenomics it increases linearly with the number of sequences, it increases exponentially with the number of PTMs considered in the search. This is the reason why in conventional search engines, only a few PTMs are queried at a time. To overcome this issue, multi-pass searches were proposed [PMID23919725: Xin et al. ISPTM: An Iterative Search Algorithm for Systematic Identification of Post-translational Modifications from Complex Proteome Mixtures. J Proteome Res 2013]; [PMID20082346: Tharakan et al. Data maximization by multipass analysis of protein mass spectra. Proteomics 2010]. The issue of TDC instability with respect to database size should be carefully considered when using multipass approaches for PTM identification, as it is the case when using reduced databases in proteogenomics or multipass searches in metaproteomics.

Further, the FDR associated to PTMs is different from the global FDR [doi.org/10.4310/SII.2012.v5.n1.a5: Fu. Bayesian false discovery rates for post-translational modification proteomics. Statistics and Interface 2012] and it has been proposed a separate FDR control for modified versus non-modified peptide identifications (as also proposed for variant versus non variant peptide identifications). Again, this causes a reduction in database size, which should be accounted for when choosing the appropriate method to control FDR.

Finally, the state-of-the-art approach to deal with PTMs at large scale (i.e., in proteomics analyses were results are globally assessed with an FDR control procedure) is to rely on Open Mass Search (OMS). Essentially, the idea is to increase the precursor mass tolerance parameter of the search engine, so that a PSM can be triggered despite the mass shift resulting from the PTM. Unfortunately, previous investigations of our lab (in a pure proteomics context, as opposed to the proteogenomics one) have demonstrated that TDC is also very unstable with respect to the precursor mass tolerance set [doi.org/10.1021/acs.analchem.0c00328]. Likewise, one can speculate that replacing TDC by BH (which has been mathematically demonstrated to be rather stable) should at least partially cope for the problem. However, these are so far speculations, as it is nevertheless possible that BH procedure leads to biased results in this case. Notably, BH is known to be sensitive to a specific correlation structure (namely Positive Regression Dependency on Subsets or PRDS), and if such structure shows up from the interference between the OMS and the database reduction, the FDR control may not hold anymore, leading to spurious PSMs.

Investigating this would be of prime interest, however, it raises many issues of important magnitude (among which the definition of gold standard datasets including both variant sequences and PTMs for benchmarking) and it is beyond the scope of this manuscript. Nonetheless, we recognize the interest to mention also this application of our findings and we added it in the Discussion. This is indeed another interesting example of an issue where methods of FDR control alternative to TDC, such as BH, should be considered.

Minor points

1.A.	A smaller point related to the use of transcript abundances to infer proteins. What are some limitations of the assumption that all proteins identified should have detectable transcripts? Given differences between protein and mRNA half‐lives, one could imagine some long‐lived proteins being present that do not have detectable transcripts. Does this represent a downside?

>> We agree with Reviewer 2 on this point. An intrinsic limitation of database searches in proteo(geno)mics is that one cannot work under the open world assumption: Only proteins which sequences (either canonical or transcriptome-informed) are in the database can be identified. To outstep this limitation, De Novo scoring has long been identified, but after 10 to 15 years, the performances are still below the expectations. In this context, Reviewer 1 has questioned us about the possibility to rely on spectral re-scoring methods like [PMID: 33109751], and following their advice, we have added a paragraph on the subject in the Discussion section.

1.B.	Additionally, integrating transcript sequencing data could be valuable in discerning between protein isoform expression – does their script enable this analysis?

>> We thank Reviewer 2 for this comment. Indeed, our R package allows to better discriminate which protein isoforms are most likely expressed, according to transcriptome evidence. However, its performance depends on the quality of transcriptome information, and in particular, on the precision used to establish which transcript isoforms were expressed. This in turn depends on the protocol used to generate the transcriptome (e.g., RNA-seq reads length, single- or paired-end sequencing, sequencing depth) and on the computational tool employed for the analysis. While other approaches already exist with the explicit aim of discriminating between different protein isoforms, e.g. [PMID30289715: Jeong et al. ASV-ID, a proteogenomic workflow to predict candidate protein isoforms on the basis of transcript evidence. J proteome res 2018], the main aim of our R package is instead to reduce overall ambiguity of protein identifications (from isoforms or from homologous genes) and to provide a transparent and measurable way to measure this reduction in ambiguity. The first aim is achieved by allowing the user to filter, based on transcriptomic information, proteomic identifications obtained from a conventional search against a canonical database – which is more transparent and reproducible than using a transcriptome-informed reduced database search. The second aim is achieved by calculating connected components as proxy of protein groups and providing the proportion of connected components containing only one protein and the proportion of specific versus shared peptides before and after filtering. Nonetheless, the package allows the user to visually inspect each group of proteins connected by shared peptides. For any particular protein of interest having a shared peptide, the user can thus easily identify which and how many proteins it shares the peptide(s) with. Given the identifiers of such proteins the user is able to know if it shares peptide(s) with isoforms of the same gene or with proteins from different genes or both.

2.	Overall the data is nicely presented. However, Figure 3A does not have axes labeled

>> We thank Reviewer 2, both for the comment, and for mentioning the label omission (which has been corrected).

Reviewer 3

This manuscript addresses the persistent misunderstanding of the field of the proper uses of FDR control in proteogenomics database searches. The authors demonstrate clearly that it is a mistake to associate an increase in proteomics search sensitivity with a decrease in database size. Rather, this is a statistical error due to a flaw in target decoy comparisons (TDC). They demonstrate that reducing the database size can result in insufficient number of decoys to simulate incorrect target matches accurately, causing lower scoring cutoffs for the same false discovery rate (FDR). They also show that the Benjamini-Hochberg (BH) procedure produces more conservative and stable FDR control than does TDC. They then demonstrate that reduced database searches with BH-based FDR control yield smaller protein groups and protein identifications with less ambiguity. Finally, the authors use an alternative strategy that consists of a full database tandem MS search with post-hoc removal of proteins for which no transcript was expressed, and no peptide identified. This reduces the number of ambiguous protein identifications that result from shared peptides. In summary, if this work is read and understood widely, there will be an improvement in the appropriate use of proteogenomics data in the field.

>> We thank Reviewer 3 for their very accurate summary of our manuscript, as well as for their positive evaluation.

**2^nd^ round**

**Reviewer 1**

The authors have done a good job at addressing the wide range of comments that were raised by three reviewers. Changing the focus of the paper on creating transcriptomics-informed databases for eukaryotes (also reflected by a change in the title and many changes throughout the manuscript) has made the message much clearer. The authors have increased the number of data sets analyzed and search engines employed, which supported the conclusions.

I support acceptance of the revised manuscript in Genome Biology.

**Reviewer 3**

The authors have nicely answered concerns from the prior review. My only comment is that in the Results and Discussion sections there are some rather long and dense paragraphs that span several pages. In the final editing before publication I would suggest looking for some ways to break up some of the long paragraphs into shorter paragraphs that will be more accessible to readers.
